# Is the presence of Modic changes associated with the outcomes of different treatments? A systematic critical review

**DOI:** 10.1186/1471-2474-12-183

**Published:** 2011-08-10

**Authors:** Rikke K Jensen, Charlotte Leboeuf-Yde

**Affiliations:** 1 Research Department, Spine Centre of Southern Denmark, Clinical Locomotion Network, Hospital Lillebaelt, Middelfart, Denmark; 2 Institute of Regional Health Services Research, University of Southern Denmark, Odense, Denmark

## Abstract

**Background:**

Modic changes (MCs) have been identified as a diagnostic subgroup associated with low back pain (LBP). The aetiology of MCs is still unknown and there is no effective treatment available. If MCs constitute a specific subgroup of LBP, it seems reasonable to expect different effects from different treatments. The objective of this systematic critical literature review was therefore to investigate if there is evidence in the literature that the presence of MCs at baseline is associated with a favourable outcome depending on the treatment provided for LBP.

**Methods:**

The databases MEDLINE and EMBASE were searched for relevant articles from 1984 to December 2010. A checklist including items related to the research questions and quality of the articles was used for data extraction and quality assessment. Of the 1650 articles found, five (six studies) were included in this review but because the studies were so heterogeneous, the results have been reported separately for each study.

**Results:**

The treatments studied were: lumbar epidural steroid injections (n = 1), lumbar intradiscal steroid injections (n = 2), lumbar disc replacement (n = 1), fusion surgery (n = 1) and exercise therapy (n = 1). One of the two studies investigating treatment with intradiscal steroid injections and the study investigating fusion surgery reported that MCs were positively associated with the outcomes of pain and disability. The other study on lumbar intradiscal steroid injections and the study on lumbar epidural steroid injections reported mixed results, whereas the study on lumbar disc replacement and the study on exercise therapy reported that MCs were not associated with the outcomes of pain and disability.

**Conclusions:**

The available studies on the topic were too few and too heterogeneous to reach a definitive conclusion and it is therefore still unclear if MCs may be of clinical importance when guiding or prescribing the 'right' treatment for a patient with LBP.

## Background

Low back pain (LBP) is a large problem in the Western world. It has considerable financial consequences both for the individual person and for society. Currently, less than 15% of patients seeking care for LBP are thought to have specific causes of LBP [1], leaving the remaining 85% classified as having 'non-specific LBP'. Previous studies of various types of treatment show only little or no treatment effects in patients with non-specific LBP [2]. One explanation for this could be that non-specific LBP is a symptom caused by several different pathologies, thereby dividing patients into different pathological subgroups. If this were the case, it would not be surprising if the effect of treatment were often unimpressive, as these unidentified subgroups would be treated without any evidence-based rationale. Therefore, more knowledge on different methods of subgrouping of patients in relation to indications for various treatment approaches would be very helpful, as it would improve the possibilities for a more targeted treatment approach.

One such possible subgroup, Modic changes (MCs), have recently been associated with LBP [3]. MCs appear to be a stage of the disc degeneration process [4-7]. They were first defined in the literature by Modic et al. [8] who described two types (type I and II) of signal changes visible on magnetic resonance imaging (MRI). This research group also made a histological examination of the findings which revealed fissured endplates and vascular granulation tissue adjacent to the endplate in type I, and disruption of the endplates as well as fatty degeneration of the adjacent bone marrow in type II. Modic et al. also described a third type as corresponding to sclerosis seen on radiographs [9]. Although it is uncertain whether 'normal' disc degeneration is associated with LBP, an association between MCs and LBP was found in a systematic review by Jensen et al. who reported a positive association in seven out of ten studies with odds ratios from 2.0 to 19.9 [10].

There are two main theories as to why MCs develop - a biomechanical theory and an infection theory. The first theory is that MCs are caused by mechanical stress [11]. The degeneration of the disc leads to changes in the mechanical conditions in and around the disc [4,12]. Improper loading and shear forces then cause micro-fractures of the endplate resulting in inflammation in the vertebral endplate and the adjacent bone marrow [13,14]. Jensen et al. found that persons from the Danish general population with disc degeneration, bulges or herniations had twice the odds of new endplate changes (MCs) over four years compared with persons with normal disc contours or no degeneration [15], and Albert et al. found that among patients with disc herniation at baseline 17% had developed new MCs type I at 14-months follow-up at the same vertebral level as the previous herniated disc [16].

The second theory is that the inflammation and oedema in the vertebral endplate are caused by a bacterial infection in the associated disc [11]. Following a disc herniation, new capillarisation and inflammation occur which are thought to be a 'port' for anaerobic bacteria to enter the disc. Sterling et al. [17] found the presence of low virulent bacteria in 53% of the disc material harvested from surgery on herniated discs whereas Carricajo et al. [18] found only 7%, and argued that the presence of bacteria was due to contamination of the disc samples. The debate about the presence of bacteria in MCs type I is ongoing as Wedderkopp et al. [19] found no trace of anaerobic bacteria in 24 biopsies taken from vertebrae affected by MCs type I. However, Ohtori et al. [20] found that 4 patients out of 71 with MCs type I at baseline developed clinical symptoms of pyogenic spondylitis over a two-year period, and that 3 of those patients had a pyogenic infection confirmed with a biopsy.

If MCs constitute a specific subgroup of LBP, one would expect different outcomes with different treatments for this condition. However, this would depend on the etiology of this pathology, which remains contentious. If the mechanical theory is correct, one would expect alleviation of symptoms with rest, because immobility might be necessary to heal any micro-fractures, whereas vigorous weight bearing exercise might prevent micro-fracture healing. Similarly, a good outcome might also be expected from fusion surgery, because fusion may neutralize the biomechanical dysfunctions in the vertebral segment [21]. Perhaps for this reason, some surgeons are of the opinion that the presence of MCs is a good indication for fusion surgery [22]. In contrast, if the bacterial theory is correct, the outcome should be favourable with antibiotic treatment [23].

In other words, the presence of MCs may have an impact on outcome, either positively or negatively, for various types of treatments. In order to synthesise the evidence, we performed a systematic critical literature review. The objective was to investigate if the presence of MCs at baseline is associated with outcomes from different kinds of treatments for LBP.

## Methods

### Search strategy

The databases MEDLINE and EMBASE were searched using the following MeSH terms and/or as free text: 'MRI', 'vertebral endplate' and 'lumbar spine'. See additional file 1: MEDLINE search strategy. The first author searched the databases, assisted by a research librarian. The search period was from 1984 to December 2010. Our search was restricted to the period after 1984, because MRI was not commonly used in clinical settings before that time and consequently MCs were not diagnosed prior to that. In addition, reviews and reference lists were searched for further references, and experts were contacted for any additional references.

### Inclusion criteria

Articles were considered for inclusion if they were original articles from peer reviewed scientific journals published in English, French, German, Spanish, Swedish, Norwegian or Danish. The inclusion criteria were:

#### 1. Participants

Studies of living human adults diagnosed with LBP, with endplate changes (MCs) described at baseline and who received a described treatment and were not diagnosed with malignancy, tuberculosis, any type of traumatic fractures, or systemic inflammatory disease.

#### 2. Interventions

Any intervention or combination of interventions targeting LBP.

#### 3. Outcome

Any clinical outcome, including (but not restricted to) pain, disability and return to work.

#### 4. Study design

Prospective studies and retrospective studies, with not less than 50 participants at baseline, providing it were possible to extract relevant data to compare treatment outcomes either 1) in patients with or without MCs, or 2) in patients with different types of MCs.

### Definition of checklist items

Relevant articles were reviewed according to a checklist in which the authors defined a set of criteria consisting of descriptors, quality items and study results that were considered essential for this review. A checklist that included the items described below was devised, tested and improved before being used for data extraction.

#### 1. Descriptive items

• Study characteristics: aim, study design and description of MCs at baseline.

• Participants and treatment characteristics: age, sex, number of participants, drop-out rates, origin of population, disease characteristics and type of treatment.

• Method: type of recruitment procedure, time and number of follow-ups and outcome measures used.

#### 2. Quality items

• No fixed set of generally accepted quality criteria were found that suited this type of literature review and therefore quality criteria were chosen in consideration of factors important for a systematic review of prediction of treatment outcome. We concentrated on the following issues: 1) the generalisability of the study sample, and 2) the trustworthiness of both the MRI findings of MCs and the outcome variables. A systematic evaluation was conducted regarding eight specific quality check-list items applied to each study forming the basis for an overall interpretation of the credibility of the study results, see Table 1. The cut-points for credibility were arbitrarily defined as percentages and divided into 3 categories: 0-50% was considered 'Unacceptable', 51-75% 'Fair' and 76-100% 'Good'.

**Table 1:** The eight questions of the quality score

Reporting on drop-out:
1) Drop-out rate reported
2) Drop-out rate accounted for
3) Analysis made, to see if the responders looked like the non-responders

**Reporting on validity:**

4) Did they attempt to assure or check validity of main outcome measures?

**Reporting on MRI evaluation:**

5) Reproducibility of evaluation protocol
6) Competent evaluator
7) Standardized protocol used
8) Blinding

#### 3. Results

• Conclusions on whether MCs were found to be associated positively, negatively, or at all, with treatment effect.

### Review process

**Figure 1:**
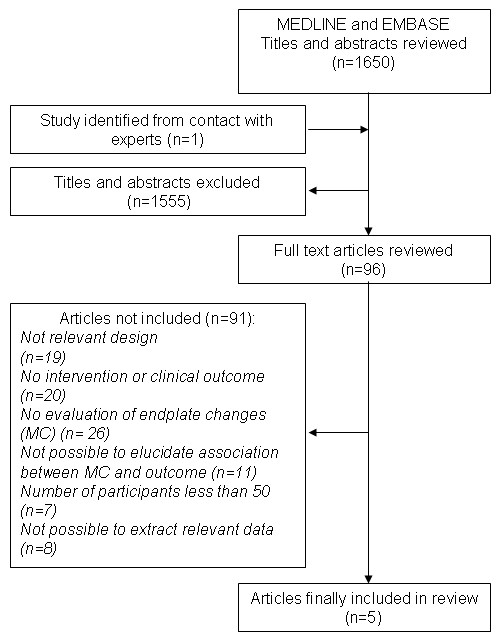
**Flowchart of review process**.

In all, 1650 references were obtained and examined by the first author on title and abstract according to the inclusion criteria. Articles written in French were evaluated by the second author. Of the 1650 references, 96 articles were retrieved in full text as hard copy and further screened by the first author according to the inclusion criteria. Six studies reported in five articles were found to meet the inclusion criteria and were independently assessed by the two authors following the checklist. The first author compared the pair of completed checklists for consistency for each article. In the case of inconsistencies, consensus between the two authors was reached through discussion. The flowchart of the review process can be seen in Figure 1.

## Results

### 1. Description of studies

The six studies included in the review were all outcome studies, published in English, explicitly designed to investigate associations or predictors of treatment effect. All studies included both men and women, with the reported mean age between 41 and 52 years, and all the patients were recruited from secondary care although the procedure for recruitment differed. One study used advertising [24], four used consecutive patient recruitment [25-27] and one did not report the procedure [28].

The treatments studied were: lumbar epidural steroid injections [25], lumbar intradiscal steroid injections [25,28], lumbar disc replacement [27], fusion surgery [26] and exercise therapy [24].

### 2. Quality assessment

In general, the quality of the studies was unacceptable to fair. All studies reported their drop-out rates but it was mostly unclear how the drop-outs were accounted for and if the patients who dropped out were different from those remaining. A reference for the validity of outcome measures was mostly reported, but only a few of the studies reported on reproducibility of their MRI evaluation protocol, information on the evaluator, blinding (if relevant) and if standardised MRI protocols were used. The quality score of each individual study is shown in Table 2.

**Table 2:** The quality of the studies according to the quality score

First author and type of treatment	1	2	3	4	5	6	7	8	Total (%)
Buttermann [25] Epidural steroid injection	+	+	-	+	-	-	-	-	3 (38%)
Buttermann [25] Intradiscal steroid injection	+	+	-	+	-	-	-	-	3 (38%)
Fayad et al. [28] Intradiscal steroid injection	+	+	-	-	+	+	+	-	5 (63%)
Siepe et al. [27] Disc replacement	+	+	-	+*	-	-	-	-	2.5 (31%)
Esposito et al. [26] Fusion surgery	+	NA	NA	+*	-	+	+	-	3.5 (58%)
Kleinstück et al. [24] Exercise therapy	+	+	+	+	+	+	+	+	8 (100%)

The numbers from 1-8 refer to the corresponding question in Table 1. Each question was scored positive (+) if the item was fulfilled, negative (-) if the item was not fulfilled and (NA) if not applicable. *Only reported for one of two outcomes and therefore counted as 0.5 in the overall score.

### 3. Results of the review

The outcome measures of pain and disability were reported in all included studies. We therefore chose to use only pain and disability as outcome measures in this review. Because the studies were so therapeutically and methodologically heterogeneous, results are reported separately for each article.

#### Lumbar epidural steroid injections

Buttermann [25] investigated the efficiency of epidural steroid injections in patients with chronic degenerative disc disease. We considered the quality of the data to be unacceptable. Patients with MCs type I at baseline had a statistically significant greater improvement in disability at 3 and 6 months but not at 12 and 24 months compared with patients without MCs or with other types of MCs. No difference in improvement was found in back pain at any time point. As the results were only reported in graphs, quantification of the exact differences was not possible.

#### Lumbar intradiscal steroid injection

In the same article, Buttermann [25] reports on lumbar intradiscal steroid injections in those patients who did not show any effect of treatment with the spinal epidural steroid injections given in the first study. We considered the quality of the data to be unacceptable. The author reports a significant difference between the groups at baseline in disability but not in pain. Thereafter, patients with MCs type I had a significantly greater improvement at 3, 6, 12 and 24 months in disability, and at 3 and 6 months in pain, compared with the other patients. The results are shown as graphs, so again, quantification of the differences was not possible.

Also, Fayad et al. [28] investigated the effect of lumbar intradiscal steroid injections in patients with chronic discogenic LBP and MCs. We considered the quality of the data to be fair. They found that patients with MCs type I and also those with mixed type but predominantly MCs type I had a significantly higher reduction in pain at 1 month but not at 3 and 6 months compared with the reduction in pain in patients with mixed types but predominantly MCs type II. There was no significant difference in disability between different types of MCs.

#### Lumbar disc replacement

Siepe et al. [27] tested the effect of total lumbar disc replacement in patients with LBP and degenerative disc disease. We considered the quality of the data to be unacceptable. The presence of MCs did not have any significant influence on the overall outcome compared with patients without MCs, when measured at 3, 6, 12, 24 or 36 months.

#### Fusion surgery

Esposito et al. [26] tested the effect of lumbar fusion in patients with chronic discogenic LBP in a prospective study. We considered the quality of the data to be fair. They found that patients with MCs type I, MCs of mixed type I and II and those without MCs improved significantly in pain and disability at a mean follow-up time of 14 months. Patients with MCs type II did not improve significantly. An analysis of the difference in improvement between groups was not reported. As the results are only shown in graphs, quantification of the difference was also not possible.

#### Exercise therapy

In a prospective study, Kleinstück et al. [24] tested the effect of exercise therapy in patients with chronic nonspecific LBP. We considered the quality of the data to be good. They found that MCs did not significantly predict a poorer outcome in pain and disability immediately after end of treatment at 3 months or at the 12 months follow-up.

For a summary of the results, see Table 3. For details of each study see additional file 2: Details of the six included studies.

**Table 3:** Association between type of MCs at baseline and the outcome in pain and disability

Type of treatment and subgroups	Outcome measures	Association at follow-up (months)		
		1	3-6	12-24

Epidural steroid injections [25]	Pain (VAS)		-	-
MCs type I vs. type II, III and non-MCs	Disability (ODI)		+	-

Intradiscal steroid injections [25]	Pain (VAS)		+	-
MCs type I vs. type II, III and non-MCs	Disability (ODI)		+	+

Intradiscal steroid injections [28]	Pain (VAS)	+	-	
MCs type I vs. type II	Disability(QDS)	-	-	

Disc replacement [27]	Pain (VAS)	-	-	-
MCs vs. non-MCs	Disability (ODI)	-	-	-

Fusion surgery [26]	Pain (VAS)			+
MCs type I vs. type II and non MCs	Disability (JOA)			+

Exercise therapy [24]	Pain (VAS)		-	-
MCs vs. non-MCs	Disability (RM)		-	-

The reported association between type of MCs at baseline and the outcome in pain and disability at 1, 3-6 and 12-24 months follow-up. +: positive association, -: no association, ODI: Oswestry disability index, QDS: Quebec disability score, JOA: Japanese Orthopaedic Association score, RM: Roland Morris Questionnaire, VAS: Visual Analogue Scale.

## Discussion

The studies identified in this review were too few and too heterogeneous to reach a definitive conclusion as to whether MCs can be used to guide optimal treatment in patients with LBP. In addition, only 1 of the 6 studies was considered to be of good quality. The results of that study, which investigated the effect of exercise, were not particularly helpful, as they failed to differentiate MCs into sub-categories. Obviously, this is important, if various stages of MCs require different types of treatments.

The other studies, of varying quality that never exceeded fair, provided a confusing picture. Results varied for different types of treatments, at different times of follow-up and for different outcomes, in a manner that could not easily be interpreted. The only treatment investigated in two separate studies was lumbar intradiscal steroid injections but the results did not concur. Furthermore, results could not be obtained from enough studies to make a systematically reporting of the standardised effect size or another uniform measure meaningful.

For those reasons, it was also not possible to interpret the findings relative to the main theories on the etiology of MCs. There was not enough weight of evidence in favour of the biomechanical theory and we found no studies that could directly cast any light on the bacterial theory.

A potential weakness of this study is that it is possible that we missed some studies on this subject, even though the search was comprehensive and included several languages other than English. Also, some relevant studies may have been overlooked, as the initial screening of abstracts and titles was undertaken by only one of the authors. Furthermore, as we were unable to find any broadly accepted quality check-lists for this type of study, we designed our own. The presence of other quality criteria could have resulted in another judgment of the quality of this study, although it is unlikely this would have changed the interpretation of the results.

The weaknesses identified in this review make it relevant to comment on the need for future studies to respect certain methodological criteria. Two types of study designs would be suitable. 1) The one arm prospective outcome study with internal control groups i.e. the presence/absence of MCs. 2) A better design is the randomised controlled trial (RCT). RCTs should be conducted and reported according to general recommendations [29]. In order to study the predictive value of MCs it would be necessary to define its various types (such as type I, type II and mixed types). Also, obviously, the normal steps to avoid selection bias and bias in data interpretation must be taken.

## Conclusions

In conclusion, the studies in this review were too few, too heterogeneous and often lacking in adequate methodological rigour, to make a definitive conclusion as to if and how MCs are an indication for specific therapies for LBP. Therefore, although MCs may be associated with pain, it remains unclear if MCs are of clinical importance for prescribing treatment for a patient with LBP and more high quality research on the topic is needed. It also seems necessary to differentiate the types of MCs in any future analysis of treatment effects involving patients with MCs.

## Competing interests

The authors declare that they have no competing interests.

## Authors' contributions

RKJ participated in conception and design, carried out the data collection and the analysis, and wrote the main parts of the manuscript. CLY participated in conception, design and data analysis and made substantial contributions to the manuscript. Both authors have read and approved the final manuscript.

## Pre-publication history

The pre-publication history for this paper can be accessed here:

http://www.biomedcentral.com/1471-2474/12/183/prepub

## Supplementary Material

Additional file 1 **MEDLINE search strategy**. Details of the search strategy used in the MEDLINE database. Click here for file

Additional file 2 **Details of the six included studies**. Details on design, number of participants, number of participants with Modic changes at baseline, intervention procedure and drop-out rate of the six included studies. Click here for file
