# Supplementary material for: Is the presence of Modic changes associated with the outcomes of different treatments? A systematic critical review
Source: BMC Musculoskelet Disord. 2011 Aug 10;12:183. doi: 10.1186/1471-2474-12-183 (PMC3162945; doi:10.1186/1471-2474-12-183)
Supplement: Additional file 1 — MEDLINE search strategy. Details of the search strategy used in the MEDLINE database. [file 1471-2474-12-183-S1.PDF]

Additional file 1. MEDLINE search strategy

1. bone marrow
2. bone marrow[MeSH]
3. endplate
4. end plate
5. end-plate
6. modic
7. osteochondrosis
8. osteochondritis
9. schmorl*
10. discovertebral
11. scheuermann*
12. or/1-11
13. lumbar
14. lumba*
15. lumbalis
16. spine
17. spine[MeSH]
18. lumbosacral-region[MeSH]
19. lumbar vertebrae[MeSH]
20. lumbar vertebrae
21. or/13-20
22. Magnetic Resonance Imaging[MeSH]
23. MRI
24. Magnetic Resonance Imaging
25. MR* NOT mRNA
26. or/22-25
27. 12 and 21 and 26
