# Supplementary material for: Is the presence of Modic changes associated with the outcomes of different treatments? A systematic critical review
Source: BMC Musculoskelet Disord. 2011 Aug 10;12:183. doi: 10.1186/1471-2474-12-183 (PMC3162945; doi:10.1186/1471-2474-12-183)
Supplement: Additional file 2 — Details of the six included studies. Details on design, number of participants, number of participants with Modic changes at baseline, intervention procedure and drop-out rate of the six included studies. [file 1471-2474-12-183-S2.PDF]

Additional file 2 - Details of the six included studies

**Buttermann 2004[25]**<br>**Epidural steroid injections**	
Design	Prospective study
N (baseline)	232
% MCs (baseline)	40% type I
Procedure	Interlaminar or foraminal epidural spinal injections, all performed under fluoroscopy by a physician who specialized in spinal steroid injections. In the MCs type I group the number of injections given was one to three, typically one week apart. The number and frequency of injections were not reported for the comparative group.
Drop-out rate	51% in MCs type I group; 60% in comparative group
**Buttermann 2004[25]**<br>**Intradiscal steroid injections**	
Design	Prospective study
N (baseline)	86
% MCs (baseline)	47% type I
Procedure	The average dose of intradiscal steroid was 9.7 mg of betamethasone for the MCs type I group and 8.3 mg for the comparative group. Injections were performed under fluoroscopy by a physician who specialized in spinal steroid injections.
Drop-out rate	68% MCs type I group; 76% in the comparative group
**Fayad et al. 2007[28]**<br>**Intradiscal steroid injection**	
Design	Retrospective study
N (baseline)	74
% MCs (baseline)	50% MCs type I, 34% mixed type but predominantly MCs type I; 16% patients mixed type but predominantly type II
Procedure	Patients received a dose of intradiscal steroid. The dose was one millilitre (25 mg) of acetate of prednisolone injected under fluoroscopic control by a radiologist highly experienced in performing spinal injections.
Drop-out rate	1, 3 and 6 months was 7%, 19% and 24% respectively
**Siepe et al. 2006[27]**<br>**Disc replacement**	
Design	Prospective non-randomised study
N (baseline)	63
% MCs (baseline)	37% (type not reported)
Procedure	They used Link SB Charité III and ProDisc II implants
Drop-out rate	2%
**Esposito 2006[26]**<br>**Fusion surgery**	
Design	Prospective study

N (baseline)	60
% MCs (baseline)	37% MCs type I; 15% MCs mixed type I and II; 23% MCs type II; 25% no MCs
Procedure	They used posterior single-level instrumented artrodesis in 56 patients (or possibly 60, as the numbers do not add up), posterolateral allograft in 38 and interbody fusion with carbon-fiber composite cages filled with bone grafting in 22.
Drop-out rate	None
**Kleinsück 2006[24]**<br>**Exercise therapy**	
Design	Prospective study
N (baseline)	53
% MCs (baseline)	62% MCs (type not reported)
Procedure	Patients exercised twice a week for 3 months. The treatment was muscle reconditioning on training machines, low-impact aerobic and stretching exercises. The different training groups were considered as one group because no differences were found between the different training groups.
Drop-out rate	10%
